# Rock–paper–scissors interactions enable thermal localization

**DOI:** 10.1093/nsr/nwag286

**Published:** 2026-05-25

**Authors:** Jiaxin Li, Cheng-Wei Qiu

**Affiliations:** Department of Electrical and Computer Engineering, National University of Singapore, Singapore; Department of Electrical and Computer Engineering, National University of Singapore, Singapore

## Abstract

Rock-paper-scissors interactions enable thermal localization to advance thermal metamaterials.

Heat transport is typically regarded as a paradigmatic diffusive process governed by local gradients and intrinsic material parameters. In contrast to wave systems, where interference and phase accumulation enable rich strategies for controlling propagation, diffusion is often considered dynamically simple and difficult to structure beyond geometric design. As a result, manipulating heat has traditionally relied on spatial variation of conductivity or convection [1]. This picture has begun to change with recent developments in topological and non-Hermitian thermal physics [2], which have uncovered qualitatively new phenomena such as topologically protected edge states [3] and non-Hermitian skin effects [4], and reshaped our understanding of diffusion. In this emerging framework, local conduction and convection can be reinterpreted as effective couplings between elementary units, allowing thermal fields to exhibit spatial organization governed by underlying topology.

To maximize the functional advantages offered by topology, recent efforts have increasingly transitioned from tailoring material parameters toward engineering the interactions between meta-atoms. This evolution reflects several concurrent trends in coupling design: from reciprocal to nonreciprocal interactions, from passive implementations to actively controlled couplings, and from static coupling coefficients to dynamically tunable ones. Nevertheless, realizing these opportunities remains challenging in diffusive systems. Most existing thermal metamaterials lack flexible mechanisms for constructing directional and programmable couplings, thereby limiting access to richer topological phases and transport functionalities.

Recently, Hu and co-workers reported in *National Science Review* a remarkable progress in the field of thermal metamaterials by proposing a thermal rock–paper–scissors (RPS) lattice inspired by cyclic dominance in ecological dynamics [5]. In the RPS relation, each element prevails over one competitor while being dominated by another, forming a closed causal loop that represents the simplest cyclic interaction topology. Using active Peltier elements, this logic is translated into heat transfer: three thermal sites are coupled such that each site drives heat toward one neighbor while receiving heat from another, establishing a directional loop from 1 to 2, from 2 to 3, and from 3 to 1 (Fig. 1). Unlike conventional diffusion governed solely by temperature gradients, the direction of heat flow here is prescribed by the relations among sites. When cyclic units are arranged sequentially along a chain, the locally preferred transfer direction accumulates spatially, leading to a novel phenomenon that temperature localizes toward one boundary despite structural homogeneity.

**Figure 1.**
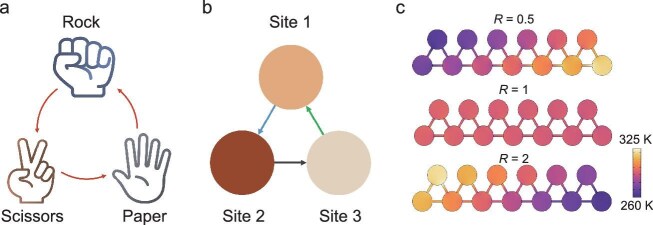
Rock–paper–scissors-inspired lattice and thermal localization. (a) The rock–paper–scissors (RPS) game illustrates cyclic dominance among three competing states. (b) Thermal analogue of the RPS motif, where three sites are coupled in a directional loop. (c) Temperature distributions in a chain for different coupling asymmetry ratios *R*, showing boundary localization of temperature. Adapted from Ref. [5].

The emergence of localization can be understood as a consequence of interaction topology. Thermoelectric pumping establishes a coupling pathway for heat exchange, thereby introducing a systematic directional bias and reshaping the eigenmode distribution. Thermoelectric elements therefore serve not only as structural connectors but also as functional regulators of transport. This upgraded coupling mechanism provides a flexible route to engineer nonreciprocal behavior beyond what is achievable in passive diffusive architectures.

This work of Hu *et al.* represents a significant advance of thermal metamaterials that highlights the emerging possibility of designing physical response through engineered structures, spanning both passive and active realizations. This may pave a novel pathway for new thermal phenomena, physical mechanisms, and advanced thermal management technologies. Looking forward, thermal metamaterials may evolve along several key directions: bio-inspired and interdisciplinary principles that translate organizational strategies observed in natural systems into material performance [6]; active metamaterial platforms enabling real-time reconfiguration of thermal modes through electrical and digital control [7]; and structure–function integrated architectures in which multiple capabilities are intrinsically embedded within compact material systems [8]. Collectively, these developments point toward a broader paradigm shift from passive media to adaptive matter, where functionality is encoded directly through designed interactions.
